# Non-linear association between AKI alert detection rate by physicians and medical costs

**DOI:** 10.1371/journal.pone.0314907

**Published:** 2025-02-26

**Authors:** Hai-bo Ai, En-li Jiang, Hai Wang, Qi Yang, Qi-zu Jin, Li Wan, Jing-ying Liu, Cheng-qi He

**Affiliations:** 1 Rehabilitation Medicine Center and Institute of Rehabilitation Medicine, West China Hospital, Sichuan University, Chengdu, P. R. China; 2 School of Rehabilitation Sciences, West China School of Medicine, Sichuan University, Chengdu, P. R. China; 3 Key Laboratory of Rehabilitation Medicine in Sichuan Province, West China Hospital, Sichuan University, Chengdu, P. R. China; 4 Dermatological Department, The Third Hospital of Mianyang, Sichuan Mental Health Center, Mianyang, China; 5 Department of Emergency Medicine, The Second Affiliated Hospital of Xi’an Jiaotong University, Mianyang, China; 6 Department of Rehabilitation Medicine, The Third Hospital of Mianyang, Sichuan Mental Health Center, Mianyang, China; Universitas 17 Agustus 1945 - Jakarta, INDONESIA

## Abstract

**Background:**

Acute kidney injury (AKI) is associated with high mortality rates and long-term adverse outcomes and significantly increases medical costs. The AKI electronic alert system built the AKI diagnostic algorithm into the medical system, along with automated collection of key indications and generation of alerts. However, the relationship between the AKI electronic alert system and medical costs is still unknown.

**Methods:**

An exploratory secondary analysis of data from a double-blinded, multicenter, parallel, randomized controlled trial to investigate the association between the AKI electronic alert system and medical costs.

**Results:**

Finally, a total of 6030 patients were enrolled in this study. Multivariate logistic regression analysis revealed that the alert group was not significantly associated with medical costs (all *p*-values >  0.05). However, the rate of alert detection by an attending physician demonstrated a notable negative correlation with medical costs; adjusted effects for direct and total costs were −126.78$ and −236.82$, respectively. The curve fitting and threshold effect analysis revealed that when the rate of alert detection by an attending physician was between 18% and 59%, each unit increase in the rate corresponded to decreases in direct cost by 363.94 (−463.34, −264.55) $ and in total cost by 698.93 (−885.78, −512.07) $. Our subgroup analysis also found a significant relationship between the rate and medical costs.

**Conclusion:**

The alert group did not significantly reduce medical costs compared to the usual care group. However, the rate of alert detection by an attending physician had a significant negative association with medical costs, and there was a threshold effect between them. When the rate was between 18% and 59%, medical costs decreased as the rate increased, and when the rate was < 18% or ≥  59%, medical costs did not decrease as the rate increased.

## Introduction

AKI is a common and increasingly prevalent condition globally, affecting both developed and developing countries [1,2]. It is associated with high mortality rates and long-term adverse outcomes, including progression to chronic kidney disease, cardiovascular diseases, and even death [3,4]. As AKI is difficult to predict in real time [5], it leads to misdiagnosis or delayed diagnosis in 25% of AKI patients in the clinic, which results in worsening of AKI disease, poor prognosis, and increased medical costs [6–8]. The AKI electronic alert system built the AKI diagnostic algorithm (KDIGO AKI criteria) into the medical system, along with automated collection of key indications and generation of alerts [9]. The automated alert system, capable of real-time prediction of AKI occurrences, is considered a potential solution to this issue [10–12]. However, there is currently inconsistency regarding the effect of alert models on the prognosis of AKI [13–16]. A BMJ RCT showed that there is no correlation between AKI alert and prognosis [16], but a secondary analysis of this study showed that the rate of AKI alert detection by an attending physician correlated with prognosis; the higher the rate of AKI alert detection by attending physicians, the better the prognosis of patients with AKI [14].

The global escalating medical costs are a major concern for both individuals and health systems [17]. From a health economics perspective, rising medical costs are associated with various factors, including population aging, the increasing burden of chronic diseases, and advances in medical technology, among others [18,19]. With the aging of the population, the elderly population is expected to increase significantly in the coming decades, which will place an additional burden on the healthcare system, as the elderly typically require a more comprehensive range of medical care [20,21]. Besides, chronic diseases, such as diabetes, cervical and lumbar pain, and high blood pressure, are another significant factor in rising healthcare costs [22]. In addition, advances in medical technology and treatments, while leading to better health outcomes, also bring higher costs [23]. Therefore, it is necessary to develop effective strategies to manage the medical costs.

As mentioned earlier in the RCT study of BMJ and its secondary analysis, we can find that associating such alert with the attending physician can improve the prognosis of patients. However, whether the AKI electronic alert system can reduce medical costs while improving patient outcomes is a topic with limited research. Therefore, we conducted a secondary analysis of a multicenter, randomized clinical trial on the AKI electronic alert system to explore whether the AKI electronic alert system can reduce medical costs while further exploring the role of the rate of AKI alert detection by an attending physician in the medical costs.

### Objective

To investigate the association between the AKI electronic alert system and medical costs.

## Methods

### Study design

This was an exploratory secondary analysis of data from a multicenter randomized clinical trial evaluating an AKI electronic alert system. From 29 March 2018 to 14 December 2019, 6030 individuals met enrollment criteria and were randomized. The data were accessed for research purposes on 10 October 2023. We had no access to information identifying individual participants during or after data collection. The trial was conducted in accordance with the principles of the Declaration of Helsinki, and two institutional review boards associated with the six study hospitals approved the study, which was deemed minimal risk. Patients were enrolled, and a requirement for informed consent was waived, as the alert was deemed unlikely to affect patient welfare, and informing patients of their diagnosis of acute kidney injury would contaminate the usual care group.

### Data source

Patient data were obtained from the online repository Dryad, with accession number https://doi.org/10.5061/dryad.4f4qrfj95. This public database archives research data and allows free access and citation [16].

### Setting

Six hospitals within the Yale New Haven Health System in Connecticut and Rhode Island, United States.

### Ethics approval and consent to participate

New ethics approval was not applicable because the original author obtained ethical approval when conducting this study. Permission to participate was also not appropriate because our review was a retrospective study of data reuse, and the patients’ messages were anonymous; our study did not contact patients.

### Inclusion and exclusion criteria

The patient inclusion and exclusion criteria were consistent with the original study [16]. Inclusion criteria: (1) The age of the inpatient was equal to or more than 18 years old. (2) Inpatient diagnosed with AKI according to the KDIGO (Kidney Disease: Improving Global Outcomes) AKI criteria; (3) For patients admitted for multiple times, only the data of the first admission were included in the analysis. Exclusion criteria: (1) patients who had previously been on dialysis; (2) patients who have end-stage renal disease; (3) patients who had an initial serum creatinine level of < 4.0 mg/L; (4) patients who are currently receiving hospice care; and (5) patients who are scheduled to undergo kidney transplantation within the next 6 months.

### AKI electronic alert system

The AKI diagnostic algorithm (KDIGO AKI criteria: A creatinine rise of 0.3 mg/dL within 48 h, or 1.5 times the lowest measured creatinine during the preceding seven days of hospitalization) was built into the medical system, along with automated collection of key indications and generation of alerts. Alerts were displayed only to individuals who had authority to change or enter new orders on behalf of the patient which included interns, residents, fellows, attending physicians, nurse practitioners, and physician’s assistants. Alerts were displayed each time the chart was opened, provided the patient continued to meet criteria for acute kidney injury. If the provider agreed or disagreed with the presence of acute kidney injury, the alert was suppressed for 48 hours for that provider. If multiple providers used the electronic health record to care for the same patient, each of them separately received the alert whenever they opened the patient chart. Patients randomized to the usual care group generated “silent” alerts, which did not display to providers [16].

### The rate of AKI alert detection by an attending physician

The rate was defined as the number of times an attending physician detects an alert, divided by the total number of alerts generated by the alert system.

### Statistical analysis

Continuous variables were summarized using mean and standard deviation. Categorical variables used counts and percentages. Univariate analysis, multivariate regression, smooth curve fitting (generalized additive modeling [24]), and threshold analysis [25] evaluated the association between the alert and the medical costs, as well as the association between the rate of alert detection by an attending physician and the medical costs. Statistical analysis was made by Empower Stats v4.2(X&Y Solutions, Inc., Boston, MA, http://www.empowerstats.com) and R v4.3.0(http://www.r-project.org, The R Foundation), which had been widely applied [26,27]. Statistical software EmpowerStats and R were used for a two-sided *P* < 0.05 significance level.

## Results

### Baseline characteristics of included patients

The study involved a total of 6030 patients, with a median age of 69.32 years, and a slightly higher proportion of males (52.18%). The majority of the patients were of other races (84.31%), while 15.69% were Black. Most of the patients were admitted for medical reasons (75.69%), and a significant proportion were recorded in urban teaching hospitals (77.91%). Approximately one-third of the patients (31.89%) were admitted to the Intensive Care Unit (ICU). The median estimated Glomerular Filtration Rate (eGFR, one of the best indices for evaluating glomerular function in normal or diseased individuals [28]) was 55.45 mL/min, and the median creatinine level was 1.01 mg/dL. The median Sequential Organ Failure Assessment (SOFA, a scoring system that assesses the degree of organ failure in critically ill patients [29]) score was 2.00, and the median Elixhauser Comorbidity Index (ECI, a composite scoring system that evaluates the prognosis of underlying diseases for hospitalized patients [30]) was 6.00. In terms of comorbidities, 37.98% of the patients had chronic kidney disease (CKD), 34.23% had Chronic Obstructive Pulmonary Disease (COPD), 41.19% had diabetes, and a significant majority had hypertension (81.99%). Furthermore, 44.08% of the patients had congestive heart failure. The composite outcome was observed in 21.14% of the patients. A nephrology consult within 14 days was noted for 23.83% of the patients. The duration of Acute Kidney Injury (AKI) was less than 2 days for 72.09%, between 2 to 7 days for 23.58%, and more than 7 days for 4.33%. Approximately half of the patients (50.73%) were alert; the median proportion of those detection by an attending physician was 0.35. The median direct cost was $10480.50 (IQR: $5392.00-$22259.25), and the median total cost was $19659.00 (IQR: $10018.50-$43074.50). (Table 1)

**Table 1:** The clinical characteristic of patients.

Characteristics	Median (Q1–Q3)/ N (%)
Age (years)	69.32 (58.11–78.72)
Sex (M/F)	3148/2882
Race, n (%)	
Other	5084 (84.31%)
Black	946 (15.69%)
Hospital recoded, n (%)	
Urban teaching	4698 (77.91%)
Suburban teaching	567 (9.40%)
Suburban non-teaching	765 (12.69%)
Duration of AKI (days)	
<2	4347 (72.09%)
2–7	1422 (23.58%)
>=7	261 (4.33%)
Medical admission, n (%)	4564 (75.69%)
eGFR (mL/min)	55.45 (35.35–83.85)
Creatinine (mg/dL)	1.01 (0.71–1.44)
Bicarbonate (mmol/L)	24.00 (21.00–27.00)
BUN (mg/dL)	28.00 (19.00–41.00)
Anion gap (mmol/L)	12.00 (10.00–14.93)
K + (mmol/L)	4.20 (3.80–4.60)
Platelet count (×1000/μL)	201.00 (146.00–266.00)
Na + (mmol/L)	138.00 (135.00–141.00)
White blood cell count (×1000/μL)	9.80 (7.20–13.60)
Hemoglobin (g/dL)	10.60 (9.00–12.30)
Elixhauser Comorbidity Index	6.00 (4.00–8.00)
CKD, n (%)	2290 (37.98%)
COPD, n (%)	2064 (34.23%)
Diabetes, n (%)	2484 (41.19%)
Hypertension, n (%)	4944 (81.99%)
Congestive heart failure, n (%)	2658 (44.08%)
Composite outcome, n (%)	1275 (21.14%)
Nephrology consult within 14 days, n (%)	1437 (23.83%)
SOFA score	2.00 (1.00–4.00)
ICU, n (%)	1923 (31.89%)
Alert, n (%)	3059 (50.73%)
The rate of alert seen by an attending physician (%)	35 (19–59)
Direct cost ($)	10480.50 (5392.00–22259.25)
Total cost ($)	19659.00 (10018.50–43074.50)

ICU, Intensive care unit; eGFR, Estimated glomerular filtration; BUN, Blood urea nitrogen; SOFA, Sequential organ failure assessment; CKD, Chronic kidney disease; COPD, Chronic obstructive pulmonary disease; AKI, Acute kidney injury.

### The results of multi-factor regression analysis

In the multi-factor analysis, adjustment variables were screened by the following method: after adding a variable to the model, if the change in effect value was ≥ 10%, the variable was included in the adjustment; if the change was < 10%, it was not included in the adjustment. The results of previous studies were also referenced to determine whether certain variables were included in the model adjustment. The Variance Inflation Factor (VIF) was used to determine whether collinearity existed between variables; if the VIF >  5, collinearity was considered to exist, and it was excluded before being included in the model adjustment. The screened variables were adjusted in two steps: model 1 for demographic characteristics, comorbidities, etc., and model 2 further adjusted for relevant laboratory tests and disease severity scores, etc., on the basis of model 1 to further validate the reliability of the findings. Based on the multivariate regression analysis presented in Table 2, the relationship between alert and direct cost as well as total cost was examined. The reference group for this analysis was the ‘Usual Care’ group. In terms of direct cost, the alert group showed a decrease compared to the usual care group across all modes. Specifically, in the crude mode, the effect value was -351.28 with a 95% confidence interval (CI) of −2282.15 to 1579.59 (P = 0.072). In Mode 1, after adjusting for age, sex, race, medical admission, ICU, Elixhauser comorbidity score, and hospital, the effect value was −251.28 (95% CI: −2143.75 to 1641.20, P = 0.79). In Mode 2, with additional adjustment for creatinine, BUN, anion gap, k + , Platelet count, Hemoglobin, SOFA score, duration of AKI, and nephrology consult within 14 days, the effect value was -138.36 (95% CI: −1938.67 to 1661.94, P = 0.88). Similarly, for total cost, the alert group also showed a decrease compared to the usual care group across all modes. In the crude mode, the effect value was −709.60 (95% CI: −4376.42 to 2957.21, P = 0.70). In Mode 1, the effect value was −473.86 (95% CI: −4049.52 to 3101.81, P = 0.80). In Mode 2, the effect value was −281.89 (95% CI: −3667.17 to 3103.40, P = 0.87). These results suggest that the alert group had lower direct and total costs compared to the usual care group, although the differences were not statistically significant. (Table 2)

**Table 2:** Multivariate linear regression analysis of the relationship between alert and direct cost as well as total cost.

Exposure	Crude mode, β (95%CI), P-value	Mode 1, β (95%CI), P-value	Mode 2, β (95%CI), P-value
Direct cost ($)			
Alert			
Usual Care	Reference	Reference	Reference
Alert	−351.28 (−2282.15, 1579.59), 072	−251.28 (−2143.75, 1641.20), 0.79	−138.36 (−1938.67, 1661.94), 0.88
Total cost ($)			
Alert			
Usual Care	Reference	Reference	Reference
Alert	−709.60 (−4376.42, 2957.21), 0.70	−473.86 (−4049.52, 3101.81), 0.80	−281.89 (−3667.17, 3103.40),0.87

Adjusted variables: Mode 1 including age, sex, race, medical admission, ICU, Elixhauser comorbidity score, hospital; Mode 2 including mode 1 + creatinine, BUN, anion gap, k + , Platelet count, Hemoglobin, Sofa score, duration of AKI, nephrology consult within 14 days.

In the multi-variable linear regression analysis, we found a significant negative association between the rate of alert detection by an attending physician and the direct cost as well as the total cost. In the crude model, for each unit increased in the rate of alert detection by an attending physician, the effect value of direct cost being seen decreased by 201.66 (95% CI: -234.36 to -168.95, P < 0.01). After adjusting for age, gender, race, medical admission, ICU, Elixhauser comorbidity score, and hospital (model 1), this effect value was -139.93 (95% CI: -178.10 to -101.77, P < 0.01). When we further adjusted for creatinine, BUN, anion gap, potassium, platelet count, hemoglobin, SOFA score, AKI duration, and nephrology consultation within 14 days (model 2), this effect value was −126.78 (95% CI: −163.26 to −90.30, P < 0.01). For total cost, we observed a similar trend. In the crude model, for each unit increase in the rate of alert detection by an attending physician, the effect value of the total cost decreased by 375.90 (95% CI: −438.02 to −313.76, P < 0.01). In model 1 and model 2, this effect value was −262.97 (95% CI: −335.08 to −190.85, P < 0.01) and −236.82 (95% CI: −305.43 to −168.21, P < 0.01), respectively. These results indicated that the rate of alert detection by an attending physician had a significant negative association with direct cost and total cost (Table 3).

**Table 3:** Multivariate linear regression analysis of the relationship between the rate of alert detection by an attending physician and direct cost as well as total cost.

Exposure	Crude mode, β (95%CI), P-value	Mode 1, β (95%CI), P-value	Mode 2, β (95%CI), P-value
Direct cost ($)	−201.66 (−234.36, −168.95), < 0.01	−139.93 (−178.10, −101.77), < 0.01	−126.78 (−163.26, −90.30), < 0.01
Total cost ($)	−375.90 (−438.02, −313.76), < 0.01	−262.97 (−335.08, −190.85), < 0.01	−236.82 (−305.43, −168.21), < 0.01

Adjusted variables: Mode 1 including age, sex, race, medical admission, ICU, Elixhauser comorbidity score, hospital; Mode 2 including mode 1 + creatinine, BUN, anion gap, k + , Platelet count, Hemoglobin, Sofa score, duration of AKI, nephrology consult within 14 days.

### The results of curve fitting and threshold effect analysis

**Fig 1:**
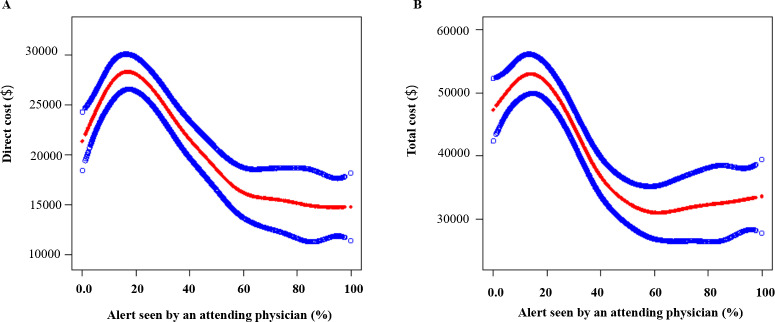
The results of curve fitting analysis. A and B illustrated the trends in medical costs as a function of the rate of alert detection by an attending physician. The x-axis in both graphs represented the percentage of alert detection by an attending physician, ranging from 0% to 100%, while the y-axis showed costs in dollars. A specifically depicted direct cost, and B showed total cost. In figure A, we saw that direct cost initially increased sharply as the alert detection by an attending physician, peaking around 18%, then steadily decreased as the rate of alert detection by an attending physician continued to climb. This curve demonstrated a non-linear relationship between the rate of alert detection by an attending physician and direct cost. The red line indicated the median trend, while the blue lines may have represented upper and lower confidence intervals. Figure B reflected a similar pattern to figure A, but on a different scale. Total cost substantially raised at lower alert percentages, reached a maximum around 18%, then progressively declined as alert rate increased. The separation between the red and blue lines was consistent with typical statistical representations of central and dispersion measures.

After adjusting for age, sex, race, admission type, ICU, Elixhauser comorbidity score, hospital, creatinine, BUN, anion gap, k +, platelet count, hemoglobin, SOFA score, duration of AKI, and nephrology consult within 14 days, a nonlinear relationship and significant threshold effect were found between the rate of alert detection by an attending physician and the direct cost as well as the total cost (P for trend < 0.01 for both). When the rate of alert detection by an attending physician was less than 18%, for each unit increased in the rate of alert detection by an attending physician, direct cost increased by 208.40 (95% CI: 7.63 to 409.16, P =  0.04) and total cost increased by 381.48 (95% CI: 4.04 to 758.91, P <  0.05). When the rate of alert detection by an attending physician was between 18% and 59%, for each unit increased in the rate of alert detection by an attending physician, direct cost decreased by 363.94 (95% CI: −463.34 to −264.55, P <  0.01) and total cost decreased by 698.93 (95% CI: −885.78 to −512.07, P <  0.01). When the rate of alert detection by an attending physician was higher than 59%, there was no significant decrease in direct cost and total cost (*P* values were 0.75 and 0.56, respectively). These results indicated that there was a nonlinear relationship between the rate of alert detection by an attending physician and the medical costs of patients, and that too low or too high proportions of alert detection were not conducive to reducing medical costs, while moderate proportions of alerts detection can effectively reduce medical costs (Fig 1 and Table 4).

**Table 4:** The results of threshold effect analysis.

The rate of alert detection by an attending physician	Direct cost ($), β (95%CI), P-value	Total cost ($), β (95%CI), P-value
<18%	208.40 (7.63, 409.16), 0.04	381.48 (4.04, 758.91), < 0.05
18% ≤ and < 59%	−363.94 (−463.34, −264.55), < 0.01	−698.93 (−885.78, −512.07), < 0.01
59% ≤	20.83 (−108.62, 150.29), 0.75	72.17 (−171.21, 315.55), 0.56
*P* for trend	<0.01	<0.01

Adjusted variables: age, sex, race, medical admission, ICU, Elixhauser comorbidity score, hospital, creatinine, BUN, anion gap, k + , Platelet count, Hemoglobin, Sofa score, duration of AKI, nephrology consult within 14 days.

### The results of subgroup analysis

**Fig 2:**
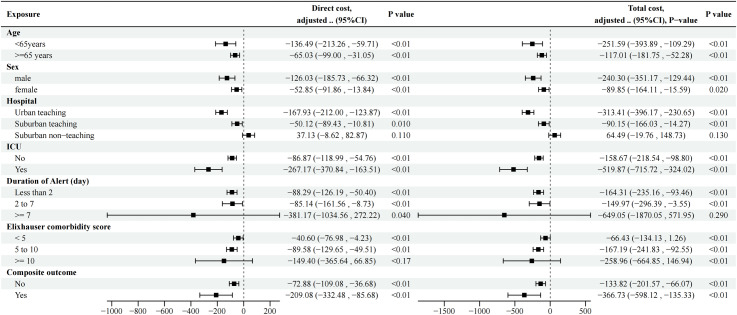
The forest plot of subgroup analysis.

In our subgroup analysis, we found a significant relationship between the rate of alert detection by an attending physician and both direct and total costs. Regardless of age (<65 years or ≥ 65 years), sex (male or female), hospital (Urban teaching or Suburban teaching), ICU status (Yes or No), duration of alert (Less than 2 or 2 to 7), elixhauser comorbidity score ( < 5 or 5 to 10), and composite outcome (Yes or No), as the rate of alert detection by an attending physician increases, both direct cost and total cost significantly decrease. However, when the duration of alert was ≥ 7 days, as the rate of alert detection by an attending physician increased, the direct cost decreased significantly, but the decrease in total cost was not significant. When the elixhauser comorbidity score was ≥ 10, as the rate of alert detection by an attending physician increased, the total cost decreased significantly, but the decrease in direct cost was not significant. In contrast, in suburban non-teaching hospitals, both direct and total costs increased with the increase in the rate of alert detection by an attending physician, although this difference was not significant (Table 5 and Fig 2).

**Table 5:** Subgroup analysis of the relationship between the rate of alert detection by an attending physician and direct cost as well as total cost.

Exposure	Direct cost, adjusted β (95%CI), P-value	Total cost, adjusted β (95%CI), P-value
Age		
<65years	−136.49 (−213.26, −59.71), < 0.01	−251.59 (−393.89, −109.29), < 0.01
≥65 years	−65.03 (−99.00, −31.05), < 0.01	−117.01 (−181.75, −52.28), < 0.01
Sex		
Male	−126.03 (−185.73, −66.32), < 0.01	−240.30 (−351.17, −129.44), < 0.01
Female	−52.85 (−91.86, −13.84), < 0.01	−89.85 (−164.11, −15.59), 0.02
Hospital		
Urban teaching	−167.93 (−212.00, −123.87), < 0.01	−313.41 (−396.17, −230.65), < 0.01
Suburban teaching	−50.12 (−89.43, −10.81), 0.01	−90.15 (−166.03, −14.27), < 0.01
Suburban non-teaching	37.13 (−8.62, 82.87), 0.11	64.49 (−19.76, 148.73), 0.13
ICU		
No	−86.87 (−118.99, −54.76), < 0.01	−158.67 (−218.54, −98.80), < 0.01
Yes	−267.17 (−370.84, −163.51), < 0.01	−519.87 (−715.72, −324.02), < 0.01
Duration of Alert (day)		
Less than 2	−88.29 (−126.19, −50.40), < 0.01	164.31 (−235.16, −93.46), < 0.01
2–7	−85.14 (−161.56, −8.73), < 0.01	−149.97 (−296.39, −3.55), < 0.01
≥ 7	−381.17 (−1034.56, 272.22), 0.04	−649.05 (−1870.05, 571.95), 0.29
Elixhauser comorbidity score		
< 5	−40.60 (−76.98, −4.23), < 0.01	−66.43 (−134.13, 1.26), < 0.01
5–10	−89.58 (−129.65, −49.51), < 0.01	−167.19 (−241.83, −92.55), < 0.01
≥ 10	−149.40 (−365.64, 66.85), < 0.17	−258.96 (−664.85, 146.94), < 0.01
Composite outcome		
No	−72.88 (−109.08, −36.68), < 0.01	−133.82 (−201.57, −66.07), < 0.01
Yes	−209.08 (−332.48, −85.68), < 0.01	−366.73 (−598.12, −135.33), < 0.01

Composite outcome: AKI progression, dialysis, or death at 14 days.

Adjusted variables (without subgroup variables): age, sex, race, medical admission, ICU, Elixhauser comorbidity score, hospital, creatinine, BUN, anion gap, K + , Platelet count, Hemoglobin, Sofa score, duration of AKI, nephrology consult within 14 days.

## Discussion

This study explored the relationship between the AKI electronic alert system and medical costs. We found that the alert group had no association with direct cost and total cost. However, the rate of alert detection by an attending physician had a significant negative association with direct cost and total cost. Further curve fitting and threshold effect analysis found that it does not reduce medical costs when the rate of alert detection by an attending physician was less than 18% or higher than 59%, while the rate of alert was between 18% and 59% can effectively reduce medical costs.

AKI is a syndrome encompassing a wide variety of aetiologies and pathophysiologic processes leading to decreased kidney function [8,31]. In low-resource settings, AKI recognition, diagnosis, and treatment initiation are often delayed or inadequate, leading to avoidable increases in mortality, severe complications, and costs [32]. The AKI electronic alert system automatically collects key indicators and generates alerts based on KDIGO criteria, supporting treating physicians in making the diagnosis of AKI and treating it appropriately [33]. In the hospital setting, randomized trials have shown the efficacy of alerts to reduce drug interactions [34], increase the rate of venous thromboembolism prophylaxis [35], and improve the rates of various other preventive measures to influence outcomes positively [36]. However, research on the relationship between AKI electronic alert system and medical costs is limited.

The medical costs of AKI patients are affected by various factors, including whether or not they are on dialysis, the progression of their disease, and the length of their hospitalization [37,38]. In particular, medical costs are significantly higher for AKI patients who require dialysis [39]. Additionally, AKI patients are often hospitalized for longer periods and require more supervision and treatment, which also leads to higher medical costs [40].

We first explored the relationship between the AKI electronic alert system and medical costs and found that the alert group did not significantly reduce medical costs compared to the usual care group, which aligns with the original study’s conclusion that the alert system did not improve patient outcomes, including the progression of acute kidney injury, dialysis, or death [16]. The progression of acute kidney injury and dialysis, both of which can increase medical expenses, may be one of the reasons that the alert group does not reduce medical costs. Additionally, the AKI electronic alert system impacted process measures, including increased intravenous fluid drip orders, urinalysis, and subsequent creatinine measurement [16], all of which can lead to increased medical costs.

Although the AKI electronic alert system did not significantly reduce medical costs, further analysis of the relationship between the rate of alert detection by an attending physician and medical costs we found that when the rate of alert detection by an attending physician was in the appropriate range (18%–59%), the higher the alert detection rate, the lower the medical costs. In the existing healthcare system, only the attending physician provided a systematic approach to AKI treatment [14]. Early detection of AKI progression might improve the patient’s prognosis by changing the dosage of medication properties, avoiding nephrotoxicity, and paying attention to fluid balance, which requires the attending physician to develop a systematic and comprehensive treatment strategy [7]. Research has found that the higher the rate of AKI alert detection by attending physicians, the better the prognosis (14-day AKI progression, 14-day dialysis, 14-day mortality, and discharge to home) of patients with AKI [14]. Preventing AKI progression, reducing dialysis, and discharge to home can all reduce medical costs, which may be why the higher the alert detection rate, the lower the medical costs. In addition, why the rate of alert detection by an attending physician was effective in reducing medical costs when it was within a certain range (18%–59%), we analyzed the possible reasons: (1) Importance of Moderate Alert Rates: When the alert detection rate by attending physicians is at a moderate level (18%–59%), it might indicate that the attending physicians are effectively identifying the AKI cases that truly require attention. In this scenario, physicians can intervene timely and prevent the progression of the condition, thus reducing the need for more expensive treatments and prolonged hospitalization. (2) Alert Fatigue: If the alert detection rate is too high (above 59%), it may lead to “alert fatigue,” where physicians become desensitized to the frequent alerts. This could result in important alerts being overlooked, impairing the ability to take effective interventions, which is not conducive to reducing medical costs. (3) Lack of attention to Alerts: A lower alert detection rate (below 18%) might suggest that attending physicians do not take alerts seriously. This could lead to missed diagnoses and missing the opportunity for timely intervention, and consequently, patients might require more extensive treatment, increasing medical costs.

In subgroup analyses, we found that when the duration of alert was ≥ 7 days, the change in total cost was not significant as the rate of alert detection by an attending physician increased. When the elixhauser comorbidity score was ≥ 10, the change in direct cost was not significant as the rate of alert detection by an attending physician increased. However, the trend of these changes was decreasing, and the reason for the statistical insignificance may be that the sample sizes of these two subgroups were relatively small. The results of our subgroup analyses also revealed an interesting phenomenon: In urban and Suburban teaching hospitals, there was a significant trend toward lower direct and total costs as the rate of alert detection by an attending physician increased. However, in Suburban non-teaching hospitals, this decreasing trend did not occur, and even patients’ medical costs showed a slight increase with the rise in the rate of alert detection by an attending physician, although this association did not reach a statistically significant level (P > 0.05). Notably, this finding echoes the observation in the original study that mortality rates increased in the alert group in Suburban non-teaching hospitals [16]. To address this discrepancy, we speculate that possible reasons include the limitations of Suburban non-teaching hospitals in terms of healthcare resources, technological capabilities, and AKI management strategies, which may result in the hospital’s inability to provide patients with timely and effective treatment options even if the alert rate of attending physicians increases, leading to progression of the condition and affecting the effectiveness of medical cost reductions [16]. At the same time, the patient population in Suburban non-teaching hospitals may have its special characteristics, such as the complexity of the condition and limited affordability, all of which may affect medical costs. Therefore, when promoting the AKI electronic Alert System in Suburban non-teaching hospitals in the future, we need to consider its applicability and implementation strategies more carefully.

## Application value of the research

The practical value of this study lies in its insights into early warning models. Firstly, our research found that as the rate of alert detection by an attending physician increases, there is heterogeneity in the changes of medical costs for patients in different hospitals. Therefore, when promoting the AKI electronic Alert System in Suburban non-teaching hospitals in the future, it is necessary to consider its applicability and implementation strategies more carefully. Additionally, the research suggested that mere warnings might not suffice to reduce medical costs. Instead, these alerts should be correlated with the attending physician to have a positive impact. In addition, this study identified a threshold relationship between the rate of alert detection by an attending physician and the medical costs, which may improve the management of the AKI electronic alert system. Medical costs may be reduced by optimizing the AKI electronic alert system to ensure that the attending physician detects alerts at the optimal range.

## Limitations of the study

The alert did not use urine output criteria, which might have meant that the study population was not fully representative of all patients with acute kidney injury. The impact of alerts on the medical behaviors of attending physicians may be a key reason why alert rates reduce medical costs. However, the specific effects of alerts on medical behaviors remain unclear and need further exploration.

## Conclusion

In conclusion, the alert group did not significantly reduce medical costs compared to the usual care group. However, the rate of alert detection by an attending physician had a significant negative association with medical costs, and there was a threshold effect between them.
